# Detection of Five Types of HPV Genotypes Causing Anogenital Warts (Condyloma Acuminatum) Using PCR-Tm Analysis Technology

**DOI:** 10.3389/fmicb.2022.857410

**Published:** 2022-05-17

**Authors:** Lixia Wu, Weifeng Wang, Jie Zhang, Xuan Wu, Yan Chen, Xiaoping Gu, Huaqing Shao, Hongsheng Li, Weiwei Liu

**Affiliations:** ^1^Department of Laboratory Medicine, Longhua Hospital, Shanghai University of Traditional Chinese Medicine, Shanghai, China; ^2^Department of Laboratory Medicine, Children's Hospital of Soochow University, Medical College of Soochow University, Soochow, China; ^3^Department of Laboratory Medicine, Central Laboratory, Shanghai Tenth People's Hospital, Tongji University of Medicine, Shanghai, China; ^4^Department of Laboratory Medicine, Shanghai Skin Disease Hospital, Tongji University of Medicine, Shanghai, China; ^5^Department of Laboratory Medicine, The Second Affiliated Hospital of Jiaxing University, Jiaxing, China

**Keywords:** human papillomavirus (HPV), melting temperature (Tm), polymerase chain reaction (PCR), condyloma acuminatum (CA), genetic testing technology

## Abstract

**Objectives:**

Condyloma acuminatum (CA) is a common sexually transmitted disease caused by human papillomavirus (HPV) infection. We established a high-throughput, simple, low-cost, and accurate HPV-typing assay (polymerase chain reaction-melting temperature [PCR-Tm] analysis) to detect HPV in CA.

**Materials and Methods:**

We detected 280 cervical scraping samples, including positive samples of HPV-6 (26), HPV-11 (12), HPV-16 (22), HPV-42 (18), HPV-43 (25), HPV-multiple (19), HPV- other type (58), and HPV-negative samples (100). All samples were compared by PCR-Tm analysis and a flow fluorescence hybridization assay. Sequencing was used to confirm the results of the PCR-Tm analysis.

**Results:**

PCR-Tm analysis was specific for each genotype (HPV-6, HPV-11, HPV-16, HPV-42, and HPV-43). The sensitivity of the PCR-Tm analysis assay for each genotype was 10^3^, 10^3^, 10^3^, 10^3^, and 10^2^ copies/reaction, respectively. Most of the 158 samples, including 58 HPV-other type positive and 100 HPV-negative samples tested by the flow fluorescence hybridization assay, were tested negative by PCR-Tm analysis. For the 122 remaining samples, 26 HPV-6, 12 HPV-11, 22 HPV-16, 18 HPV-42, 25 HPV-43, and 19 multiple HPV infections were detected through PCR-Tm analysis. In total, 25 HPV-6, 12 HPV-11, 21 HPV-16, 18 HPV-42, 25 HPV-43, and only 10 multiple HPV infections were detected by the flow fluorescence hybridization assay. The kappa coefficient for the analysis of PCR-Tm analysis and flow fluorescence hybridization assay was 0.940 (*P* < 0.0001), and the 95% confidence interval of the kappa coefficient was 90.3–97.7%.

**Conclusion:**

PCR-Tm analysis enabled the detection of HPV-6, HPV-11, HPV-16, HPV-42, and HPV-43, including single and multiple infections.

## Introduction

Human papillomavirus (HPV) is a small, double-stranded, circular DNA virus. It is classified into high-risk HPV and low-risk (LR) types based on HPV-mediated carcinogenesis (Zengin et al., 2019). Infection with high-risk HPV is highly associated with the development of cervical cancer in women, and persistent infection leads to more than 99% of cervical cancers (Jones et al., 2017). Warts are benign epithelial growths caused by HPV. Condyloma acuminatum (CA), also known as an anogenital wart, is among the most common sexually transmitted infections caused by HPV infection, especially persistent infection with LR HPV genotypes (Martinez-Mendez et al., 2018; Short et al., 2019). A two-faceted classification system for HPV-driven anogenital lesions has been established: (i) low-grade squamous intraepithelial lesions (LSILs) and (ii) high-grade squamous intraepithelial lesions (HSILs) (Darragh et al., 2013). Clavero et al. reported a good concordance between the histological diagnosis of anal HSIL and the detection of oncogenic HPV types. However, anogenital LSILs may carry foci of HSIL. Clinically diagnosed anal warts cannot be assumed to be limited to LSIL but also exist in HSIL, indicating that anal warts are susceptible to neoplastic progression (Clavero et al., 2017). Although at least 40 HPV genotypes are known to cause CA- like HPV- 6, HPV-11, HPV-16, HPV-42, HPV-43, and others (Jaworek et al., 2018), most CAs were associated with persistent infection by low-risk HPV-6 and HPV-11 (Rositch et al., 2013). The HPV infection may persist even after visible warts subside. Mechanical stimulation, injury, immunosuppression, inflammation, and other extracellular factors will affect the virus copy number in the latently infected cells, which may lead to the recurrence of warts (Doorbar, 2013). Screening for HPV infections is not able to prevent condyloma acuminata but provides the diagnosis of disease and detection of recurrence (Jaworek et al., 2018), while human papillomavirus (HPV) genotyping also has important clinical implications for the treatment of CA.

Polymerase chain reaction (PCR)-based techniques are most commonly used to detect HPV infection and determine the genotype, including conventional PCR (Jacobs et al., 1995), nested PCR (Moussavou-Boundzanga et al., 2017; Nilyanimit et al., 2018), real-time PCR (Harlé et al., 2018), multiplex PCR assays, and other PCR assays. Although nested PCR and real-time PCR offer a number of advantages over conventional PCR, such as high sensitivity and improved accuracy, these methods suffer from the same drawbacks of low throughput and being time-consuming for large-scale and multigene tests. Multiplex PCR has many advantages such as low cost, saving time, and higher throughput. Different methods are used to detect PCR products, and these methods can be divided into open-tube detection and closed-tube detection. The open-tube detection assay identifies the PCR products by electrophoresis (Caméléna et al., 2019), mass spectrometry (Ashley et al., 2018; Fernandez Asensio et al., 2018), liquid chip (hui Xu et al., 2017), sequencing (Nilyanimit et al., 2018), and dot blot hybridization (Kang et al., 2019). After opening the tube and removing the product, the disadvantages of these assays include complex equipment and procedures, high cost, and a higher false-positive rate due to laboratory contamination caused by having an open tube. Closed-tube detection assays include high-resolution melting curve analysis (Sirous et al., 2018), fluorescence melting curve analysis (Amorim et al., 2018), and fluorogenic PCR (TaqMan) assay (Troskie et al., 2019; Van der Heyden et al., 2019), all of which have the shortcoming of having low- flux detection in a single tube.

PCR-melting temperature (PCR-Tm) analysis is a single closed-tube multiplex PCR assay combining PCR and melting curve analysis. This assay needs to design some different fluorescent probes based on the base-quenched probe technology (Luo et al., 2009; Qin et al., 2014; Mao et al., 2018a,b), and design and synthesize a tag complement sequence that is complementary to the fluorescent probe. To allow a fluorescent probe to identify a series of homologous tag complements with different Tm values, we created several single -nucleotide polymorphism (SNP) sites by altering some bases in the original tag complement sequence (Wang et al., 2005). Subsequently, a series of homologous tags, which are complementary to the tag complements that were added to the upstream primer of different target genes, and finally, the target genes are amplified by PCR and detected by Tm values (Mao et al., 2018a). Thus, multiple target genes can be determined simultaneously by a single closed-tube test using one fluorescent channel, and the detection flux can be further increased by adding more tags and using additional fluorescent channels. By using PCR-Tm analysis, each target gene was identified by Tm values. The PCR-Tm analysis technique not only enables closed-tube operation but also improves the throughput and simplifies product identification. Previous research by Zhan et al. reported that the PCR-Tm analysis is believed to have the capability to identify concurrently more than 30 genes in one closed tube (Zhan et al., 2020). Therefore, we chose PCR-Tm analysis as a method for research and anticipate that it will be of great help in clinical work.

We developed a PCR-Tm analysis method to detect HPV genotypes and assess the interrater agreement with the flow fluorescence hybridization assay. Our aim was to validate the PCR-Tm analysis technique as a high-throughput, simple-operation, low-cost, and accurate method for the detection and identification of HPV-6, HPV-11, HPV-16, HPV-42, and HPV-43.

## Materials and Methods

### Study Design, Setting, and Population

We collected 280 cervical scrape samples and selected 5 HPV types, namely, HPV-6, HPV-11, HPV-16, HPV-42, and HPV-43. Hemoglobin subunit beta (HBB) and hemoglobin subunit delta (HBD) genes were selected as reference genes. One fluorescence detection channel (FAM) was deployed for PCR-Tm analysis, such that HPV-6, HPV-11, HPV-16, HPV-42, HPV-43, and HBB/D genes would be all detected in the FAM channel. Meanwhile, we designed a probe for the FAM fluorescence channel based on the base-quenched probe technology. When constructing and optimizing the PCR-Tm analysis systems for five HPV subtypes, we used plasmids cloned with five HPV DNA segments and human genome DNA samples extracted from whole blood. While using PCR-Tm analysis for clinical HPV screening, we collected cervical scrape samples that had already been tested by the flow fluorescence hybridization assay, which included single and multiple positive samples of five HPV subtypes, positive samples of other subtypes, and HPV-negative samples. To evaluate PCR-Tm analysis, we assessed the interrater agreement of the two methods.

### Clinical Sample Collection and Analysis

All 280 cervical scrape samples were collected from June 2019 to November 2021 and had already been tested by the flow fluorescence hybridization assay (Shanghai Tenth People's Hospital and Shanghai Skin Disease Hospital). To guarantee the sample quality, we performed extraction and detection as soon as possible after collecting the samples to prevent DNA degradation. Ultimately, a panel of 280 cervical scrape samples was selected, including positive samples of HPV-6 (*n* = 25), HPV-11 (*n* = 12), HPV-16 (*n* = 21), HPV-42 (*n* = 18), HPV-43 (*n* = 25), HPV-multiple (*n* = 19), HPV-other type positive (*n* = 60), and HPV-negative samples (*n* = 100). Then, we detected all clinical samples through PCR-Tm analysis and compared the results with the flow fluorescence hybridization assay. Data from flow fluorescence hybridization assays may be unreliable as there is the potential for false-positive findings due to open-tube detection. Since Sanger sequencing still represents the gold standard for molecular diagnosis, all the positively tested samples by PCR-Tm analysis were validated using Sanger sequencing (Tsingke Biological Technology, Shanghai, China). We assessed the interrater agreement of the PCR-Tm analysis assay and the flow fluorescence hybridization assay.

### DNA Extraction

Cervical exfoliated cell samples were collected by gynecologists using a specialized soft brush according to the standard operating procedure. Cervical exfoliated cells were placed in a 3-ml sample preservation medium (Tiangen Life Science, Shanghai, China) and kept frozen at −80°C until analysis. To determine the sensitivity and specificity of the PCR-Tm analysis assay, HPV DNA was extracted from all collected cervical exfoliated cell samples using the TIANamp Genomic DNA Kit (Tiangen Biotech, Beijing, China). After extraction, DNA was eluted in 50 μl of distilled water and stored at −20°C for later PCR-Tm analysis. Extracted DNA was quantified using Nanodrop 2000. The range of the nucleic acid concentration after quantifying was from 7.0 to 197.2 ng/μl.

### Primer and Probe Design for HPV Genotypes

We designed relevant primers and probes with reference to the first publication by Manos et al. (Manos et al., 1989), describing the detection and genotyping of HPV using PCR primers and probes. The HPV genome includes the early transcription region (E region, including genes *E1–E7*), the late transcription region (L region, including genes *L1* and *L2*), and the non-coding region (also known as the long control region, located between the L1 and E6 open reading frames). Specifically, HPV-6 and HPV-11 primers are located in the E6 region, HPV-16 primers are in the E1 gene, and HPV-42 and HPV-43 are in the L1 region. The lengths of the amplification are between 98 and 233 bp. Type-specific primers were designed based on conserved sequences among each HPV type with Primer Premier 5 software, and the tag and the phosphate groups were added to the 5′ and 3′ ends of the upstream primer, respectively. A FAM fluorescent channel probe based on substrate quenching probe technology was designed, which was homologous to the tag sequence. A total of 15 tag complement sequences homologous to the designed tags were evaluated using melting curve analysis (Table 1), and the appropriate tag sequences were screened and added to the 5′ end of the upstream primer of HPV to form the final tagged primers. All the primers, probes, and tag complement sequences were synthesized by Sangong Biotech Co., Ltd. (Shanghai, China). The specificity of all primers and sequences was verified using the NCBI BLAST program (http://www.ncbi.nlm.nih.gov/BLAST/). The crucial information about the match/mismatch of the universal probe to each of the PCR amplified targets on the primers is shown in Table 2. More details on the specific genomic positions of the five pairs of HPV forward and reverse primers and the length of the amplimers are shown in Supplementary Table 1.

**Table 1:** Summary of Tm of pre-tag sequences in the FAM fluorescence detection channel.

**Name**	**Pre-tag (5' → 3')**	**Tm (**°**C)**
F1	GGGGGGTGGAAATGTATAAGCTAGGTAATGG	42
F2	GGGGGGTGGAAGTGCTTAAGGTTCGTAATGG	47
F3	GGGGGGTGGAAGTGTATAAGCAAGGTAATGG	51
F4	GGGGGGTGGAAGTCATTAAGGTTGGTAATGG	52
F5	GGGGGGTGGAAGTGTATAAGGCAGGTAATGG	55
F6	GGGGGGTGGAAGTGTATAAGGTAGGTAATGG	57
F7	GGGGGGTGGAAGTGTATAAAGTTGGTAATGG	55
F8	GGGGGGTGGAAATGTATAAGGTTGGTAATGG	55
F9	GGGGGGTGGAAGTGTATAAGGTTGGTATTGG	58
F10	GGGGGGTGGAAGTGTATAAGGTTGGTAATGG	62
F11	GGGGGGTGGAAGTGTATAAGGTTGGTCATGG	54
F12	GGGGGGTGGCAGTGTATAAGGTTGGTCATGG	53
F13	GGGGGGTGGCAGTGTATGAGGTTGGTCATGG	49
F14	GGGGGGTGGCAGTGCATGAGGTTGGTCATGG	44
F15	GGGGGGTGGCAGCGCATGAGGTTGGTCATGG	37

**Table 2:** Universal probe and mismatched targets.

**Pre-tag**	**Mismatched targets**	**Tm (**°**C)**	**Gene**
	5'-CCATTACCAACCTTATACACTTCCAC-3'	Universal probe	
	3'-CACCTTCACATATTCCAACCATTACC-5'	(inverted to reflect base pairing polarity with tags)	
F1	5'-GGGGGGTGGAAATGTATAAGCTAGGTAATGG-3'	42	HPV 6: 3 mismatches to universal probe
	3'-CACCTTCACATATTCCAACCATTACC-5'		
F2	5'-GGGGGGTGGAAGTGCTTAAGGTTCGTAATGG-3'	47	HPV 11: 3 mismatches to universal probe
	3'-CACCTTCACATATTCCAACCATTACC-5'		
F3	5'-GGGGGGTGGAAGTGTATAAGCAAGGTAATGG-3'	51	HPV 42: 3 mismatches to universal probe
	3'-CACCTTCACATATTCCAACCATTACC-5'		
F7	5'-GGGGGGTGGAAGTGTATAAAGTTGGTAATGG-3'	55	HBB/D: 1 mismatch to universal probe
	3'-CACCTTCACATATTCCAACCATTACC-5'		
F10	5'-GGGGGGTGGAAGTGTATAAGGTTGGTAATGG-3'	62	HPV 43: 0 mismatches to universal probe
	3'-CACCTTCACATATTCCAACCATTACC-5'		
F15	5'-GGGGGGTGGCAGCGCATGAGGTTGGTCATGG-3'	37	HPV 16: 5 mismatches to universal probe
	3'-CACCTTCACATATTCCAACCATTACC-5'		

*The crucial information about the match/mismatch of the universal probe to each of the PCR-amplified targets on the primers was demonstrated*.

### Construction and Optimization of PCR-Tm Analysis Systems

We set up two sets of PCR reaction systems. The first PCR reaction system mix contained 10 × PCR buffer without Mg^2+^ plus (9151AM, Takara), 25 mM MgCl_2_ (9151AM, Takara), 4 × 2.5 mM dNTPs (R001A, Takara), and Taq DNA polymerase (R001A, Takara), which was used to determine the Tm values of the tag complement. The second PCR reaction used a hot-start PCR kit, including 10 × PCR buffer without Mg^2+^ plus (9151AM, Takara), 25 mM MgCl_2_ (9151AM, Takara), hot-start Taq DNA Polymerase (R007A, Takara), and 4 × 2.5 mM dNTPs (R007A, Takara). Quantitative real-time PCR was conducted on a Roche LightCycler 480 real-time PCR system (Roche Diagnostics GmbH, Switzerland).

The procedures for the PCR-Tm analysis were comprised of two parts: PCR and melting curve analysis. The PCR procedure consisted of an initial heating step at 95°C for 10 min, followed by 40 cycles of 95°C for 10 s, and 60°C for 30 s. At the end of each complete PCR run, a melting curve analysis was performed to reveal the Tm values of the PCR product. Melting curve analysis comprises an initial fluorescence acquisition step of heating at 30°C for 4 min. The temperature was subsequently increased from 30 to 80°C (ramp rate of 0.1°C/s), and the fluorescence signal was collected continuously, finally followed by a cooling step at 40°C for 30 s.

The PCR-Tm analysis system was optimized and modified based on four parts: the concentration of each untagged primer, each tagged primer, FAM probe, and Mg^2+^. To optimize the untagged primer concentration of each HPV type, different concentrations (0.08, 0.12, 0.20, 0.24, and 0.28 μM) were tested, while the other reaction systems remained unchanged. We used different concentrations of each tagged primer (0.02, 0.04, 0.06, and 0.08 μM) for the optimization test. We changed the probe concentrations (0.08, 0.12, 0.16, and 0.20 μM) to optimize the FAM probe concentration. Ultimately, optimization of the Mg^2+^ was performed by varying the concentrations (1.0, 1.5, 2.0, and 2.5 mM) and finally determining the PCR-Tm analysis system for the following experiments.

### Specificity and Sensitivity of PCR-Tm Analysis

All plasmids, including HPV-6 plasmid, HPV-11 plasmid, HPV-16 plasmid, HPV-42 plasmid, HPV-43 plasmid, and HBB/D plasmid, were constructed by inserting the HPV DNA amplicons of each HPV type and HBB/D DNA amplicons into pUC57 (JinsiruiBio, Nanjing, China). The concentration of all the original plasmid solutions was 10^12^ copies/μl. The original plasmids were serially diluted 10-fold (10^12^ copies/μl to 10^2^copies/μl) and stored at −20°C. Serial 10-fold dilutions of the plasmids were used to help validate the sensitivity and specificity of the PCR-Tm analysis assay. Specificity experiments used HPV-6, HPV-11, HPV-16, HPV-42, HPV-43, and HBB/D plasmids and a multiple-mixture comprised equal concentrations (10^6^ copies/μl) of six plasmids as templates to confirm that PCR-Tm analysis was HPV type-specific and did not cross-react. To assess the sensitivity of the PCR-Tm analysis assay, 10-fold serial dilutions of each plasmid (10^7^ copies/μl to 10^2^ copies/μl) standard were prepared as templates. Each experiment was performed in triplicate to ensure consistency.

### Statistical Analysis

To assess the interrater agreement between the PCR-Tm analysis assay and the flow fluorescence hybridization assay, we calculated the agreement ratio, 95% confidence interval, and Cohen's kappa coefficient using SPSS version 24.0 (IBM Corporation, USA).

## Results

### Testing the PCR-Tm Analysis System

**Figure 1:**
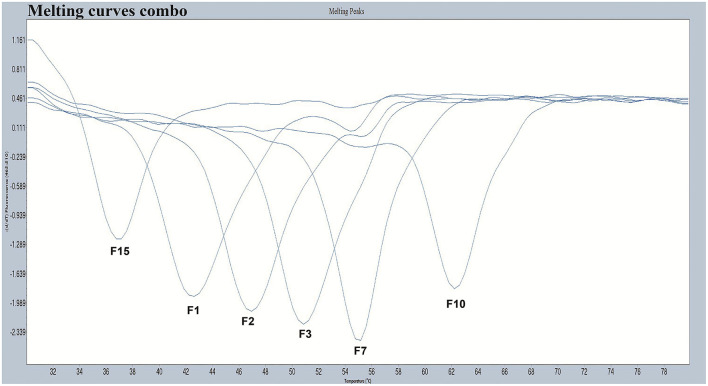
The melting curves of the chosen five pre-tag sequences in the FAM channel. Melting temperatures of F1, F2, F3, F7, F10, and F15 were 42°C, 47°C, 51°C, 55°C, 62°C, and 37°C, respectively. All the melting curves obtained in the FAM channel were distinguishable when presented together in one picture.

We designed a probe for a FAM fluorescence channel and 15 tag complement sequences. The Tm of each tag complement sequence was confirmed by a melting curve analysis. When the difference in Tm between two adjacent tag complement sequences is small, it is difficult for non-professionals to judge whether there is a dual infection in PCR-Tm analysis responses. When taking into consideration the clinical infections by multiple genotypes, based on the principle that the difference in Tm is more than 3°C, we chose six tag complements, including F1, F2, F3, F7, F10, and F15 used in the PCR-Tm analysis system, with Tm of 42, 47, 51, 55, 62, and 37°C, respectively (Table 1, Figure 1). We added the reverse complementary sequences of F1, F2, F15, F3, F10, and F7 to the upstream primer of HPV-6, HPV-11, HPV-16, HPV-42, HPV-43, and HBB/D, respectively.

### Optimization of PCR-Tm Analysis Systems

**Figure 2:**
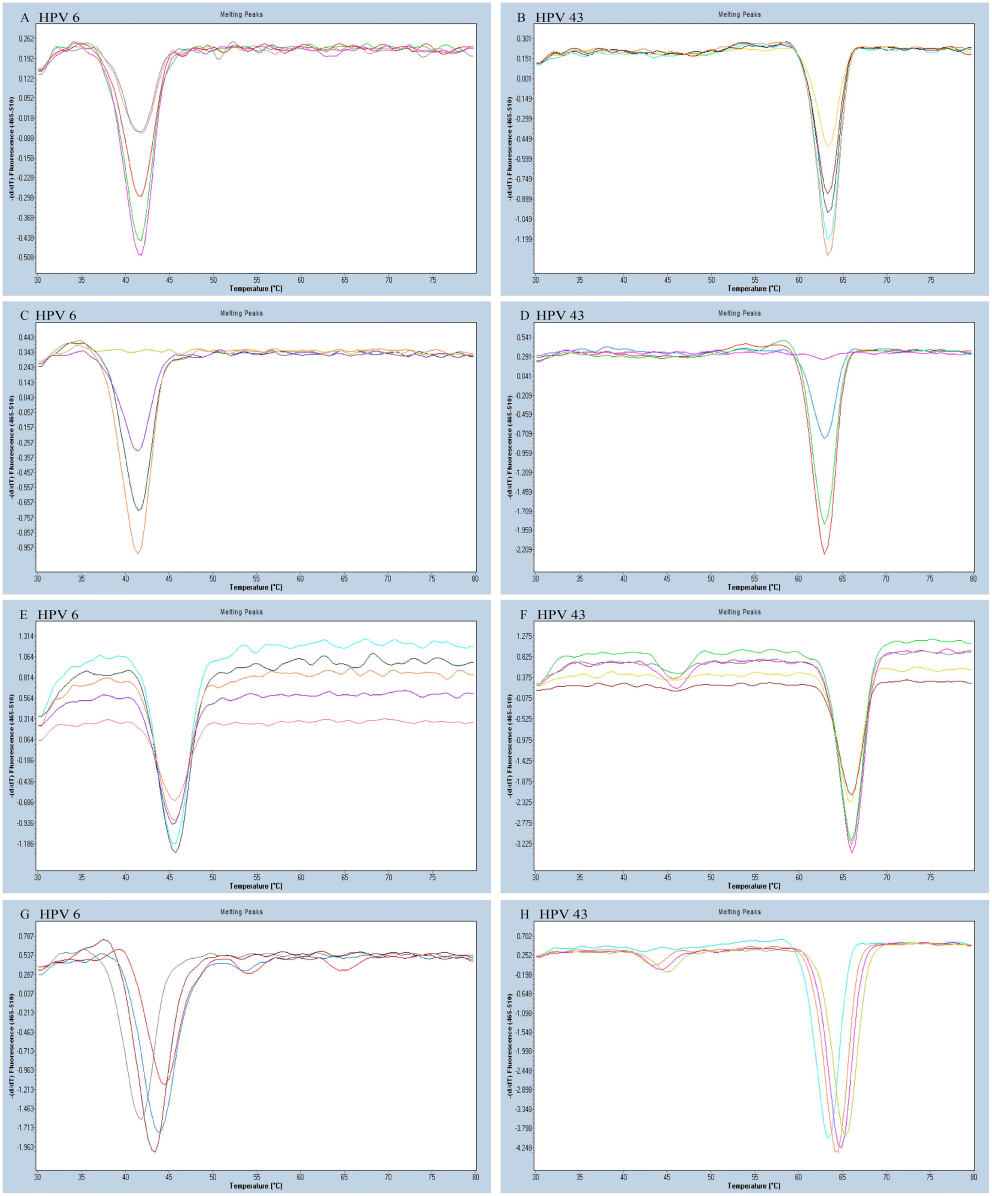
The HPV-6 and HPV-43 optimization results of a PCR-Tm analysis reaction system. **(A)** The optimum concentration of HPV-6 untagged primer was 0.24 mM (purple). **(B)** The optimum concentration of HPV-43 untagged primer was 0.28 mM (orange). **(C)** The optimum concentration of HPV-6 tagged primer was 0.04 mM (orange). **(D)** The optimum concentration of HPV-43 tagged primer was 0.04 mM (red). **(E,F)** Optimization of the concentration of FAM probe for the detection of HPV-6 and HPV-43. The optimum concentration of the FAM probe was 0.16 mM (E: gray; F: purple). **(G,H)** Optimization of the concentration of Mg^2+^ for the detection of HPV-6 and HPV-43. The optimum concentration of Mg^2+^ was 1.5 mM (G: brown red; H: orange).

As all the homologous tags and the matching FAM-labeled probe will compete for the reverse complementary sequences of the tags, this kind of competition may interfere with the melting curve analysis and potentially be more prone to false positives. To reduce the interference of competition, untagged primers must have higher concentrations than the tagged primers in the reaction. We tested different concentrations of each untagged primer including 0.08, 0.12, 0.20, 0.24, and 0.28 μM to optimize the reaction system. We altered the tagged primer concentration of each HPV type including 0.02, 0.04, 0.06, and 0.08 μM. According to the optimization results, the optimum concentrations of each tagged primer and untagged primer were HPV-6 (0.04 and 0.24 μM), HPV-11 (0.04 and 0.24 μM), HPV-16 (0.08 and 0.24 μM), HPV-42 (0.04 and 0.24 μM), HPV-43 (0.04 and 0.28 μM), and HBB/D (0.04 and 0.24 μM) (Figures 2A–D).

We varied the FAM-labeled probe concentration (0.08, 0.12, 0.16, and 0.20 μM) to look for a suitable concentration. We increased the number of probes to reduce competition and distraction from the high fluorescent background. The FAM-labeled probe concentration optimization experiment determined that the optimal amount of FAM-labeled probe is 0.16 μM (Figures 2E,F).

Increasing Mg^2+^ concentration resulted in increased specificity of PCR. However, an overly high Mg^2+^ concentration might have caused lowered enzyme activity, which may have reduced the PCR amplification efficiency. Finally, we varied the Mg^2+^ concentrations (1.0, 1.5, 2.0, and 2.5 mM) to find the optimal reaction system, and the most suitable concentration of Mg^2+^ was 1.5 mM (Figures 2G,H). The final optimized and modified PCR-Tm analysis system contained 1 × PCR buffer without Mg^2+^ plus, 1.5 mM MgCl_2_, 0.28 mM dNTPs, 2.5 U hot-start Taq DNA polymerase, 0.16 μM FAM probe, 0.24 μM of each untagged primer (0.28 μM for HPV-43), 0.04 μM of each tagged primer (0.08 μM for HPV-16), 2 μl of the sample DNA, and finally made up to a total volume of 25 μl with deionized-distilled water.

### Specificity and Sensitivity Evaluation of the PCR-Tm Analysis System

**Figure 3:**
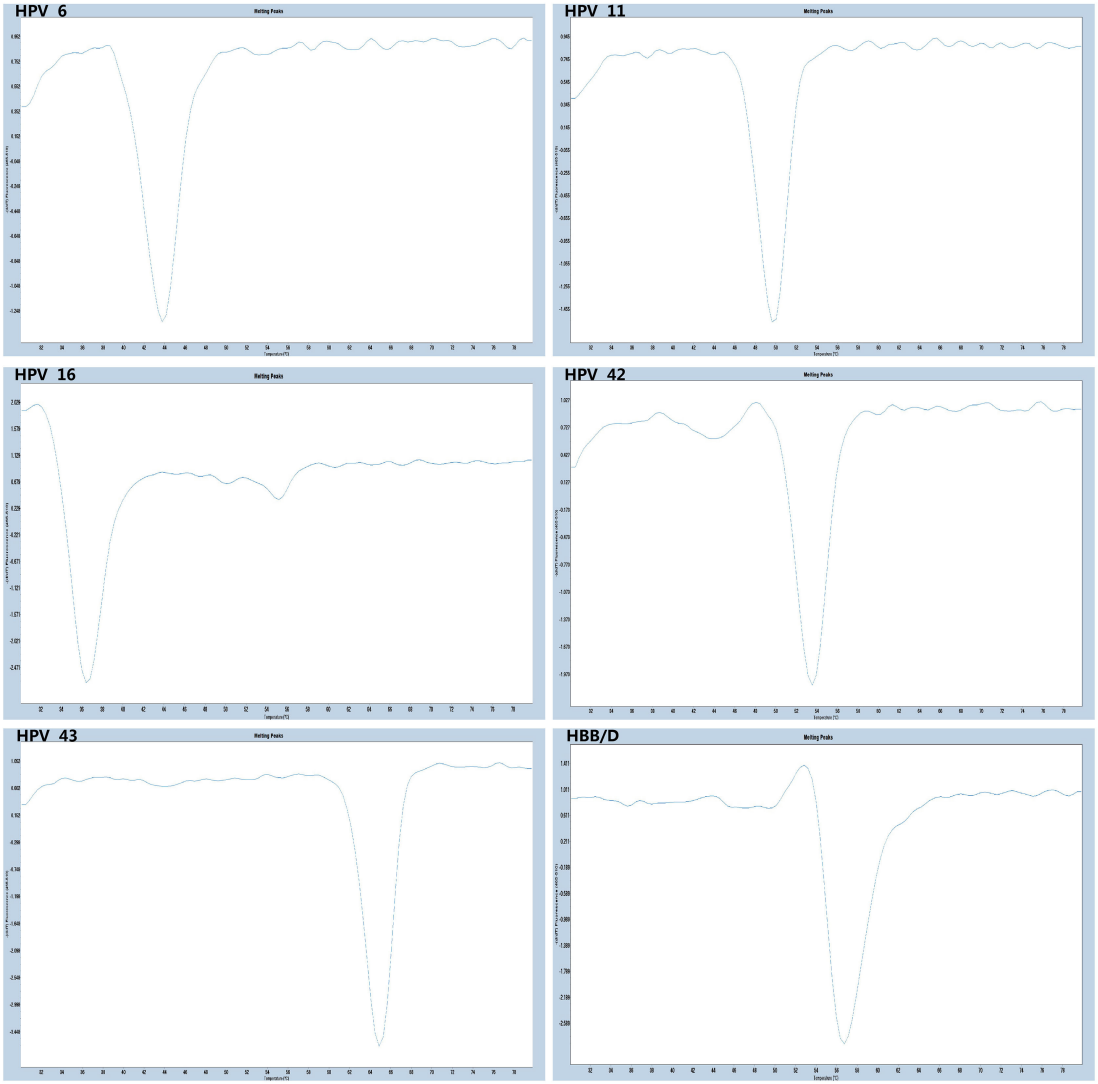
Specificity test of PCR-Tm analysis. We observed only one melting curve and no cross-reaction, and all the melting curves of HPV-6, HPV-11, HPV-42, HPV-16, HPV-43, and HBB/D were distinguishable.

**Figure 4:**
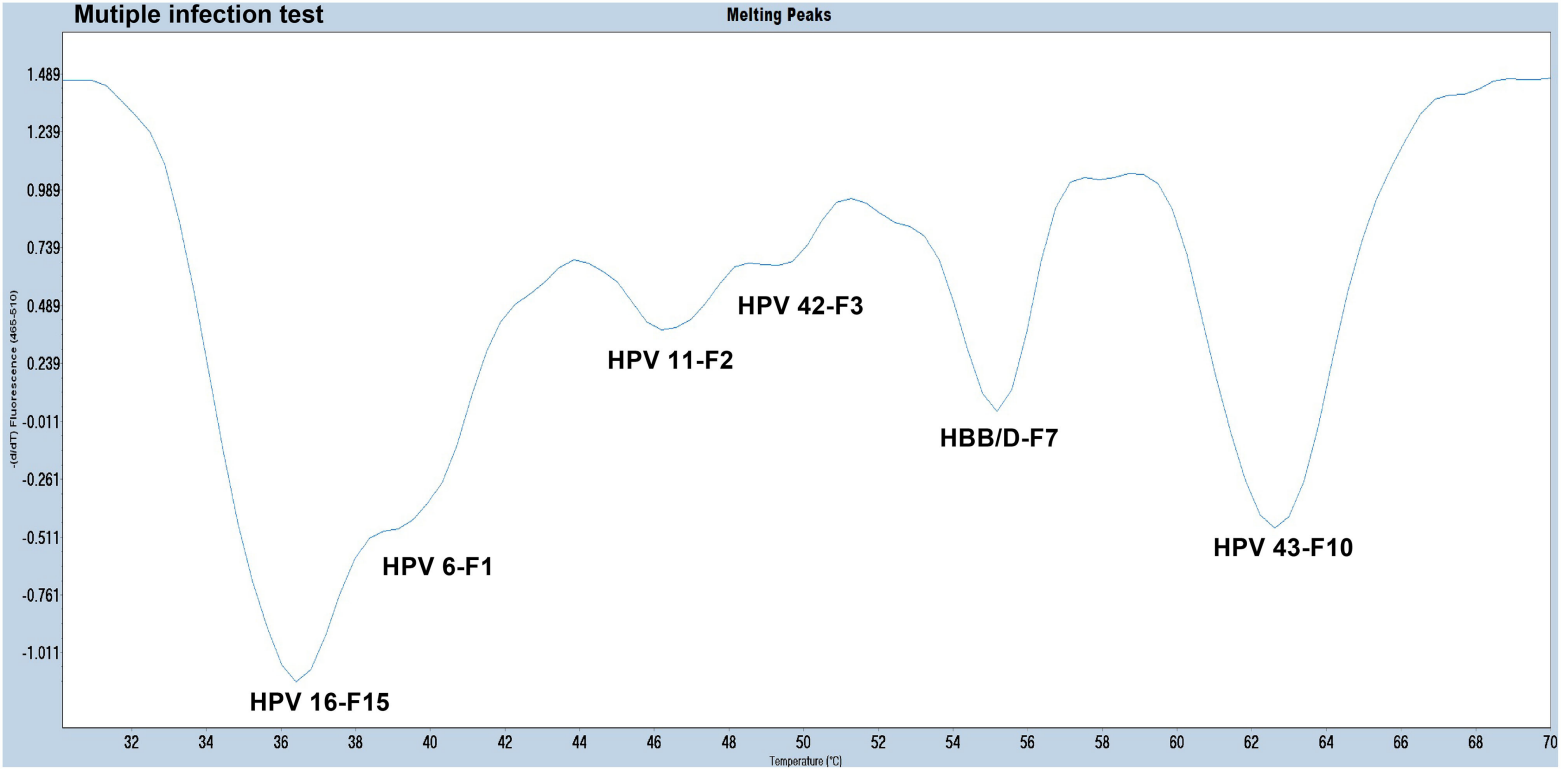
The melting curve of a multi-HPV infection test. The PCR-Tm analysis detected a 6 fold infection when a mixture of five HPV plasmids and HBB/D was used as a template.

**Figure 5:**
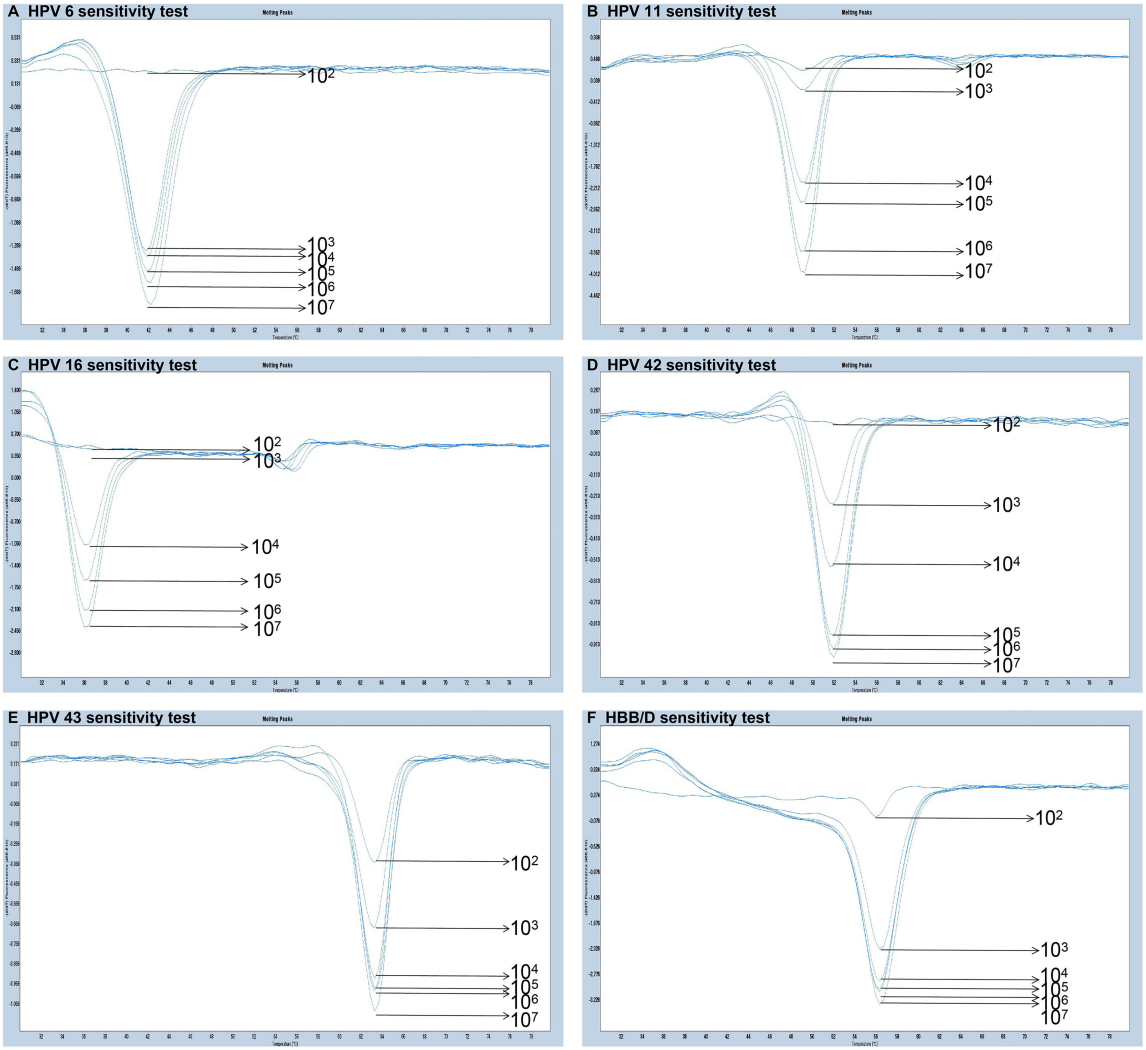
Sensitivity test of PCR-Tm analysis. **(A)** The sensitivity of HPV-6 was 10^3^ copies/reaction. **(B)** The sensitivity of HPV-11 was 10^3^ copies/reaction. **(C)** The sensitivity of HPV-16 was 10^4^ copies/reaction. **(D)** The sensitivity of HPV-42 was 10^3^ copies/reaction. **(E)** The sensitivity of HPV-43 was 10^2^ copies/reaction. **(F)** The sensitivity of HBB/D was 10^2^ copies/reaction.

The experiments performed were designed to validate the specificity of the PCR-Tm analysis assay and consisted of two parts. First, to verify the specificity of PCR primers, single HPV-6, HPV-11, HPV-16, HPV-42, HPV-43, and HBB/D plasmids were used as DNA templates. The results revealed high specificity and no cross-reactivity (Figure 3). The Tm of HPV-6, HPV-11, HPV-16, HPV-42, HPV-43, and HBB/D plasmids were 44, 49, 36, 53, 64, and 56°C, respectively. Second, a mixture of six HPV plasmids was used as a DNA template to evaluate the ability of PCR-Tm analysis assays to detect multiple infections. The PCR-Tm analysis detected a 6 fold infection when a mixture of six HPV plasmids was used as a template, but we observed that the depths of the melting curves were different from single infections (Figure 4). The sensitivity of the PCR-Tm analysis assay was evaluated using 10^2^ to 10^7^ plasmid copies of each HPV type. The cutoff sensitivities of HPV-6 (Figure 5A), HPV-11 (Figure 5B), HPV-16 (Figure 5C), HPV-42 (Figure 5D), HPV-43 (Figure 5E), and HBB/D (Figure 5F) were 10^3^, 10^3^, 10^4^, 10^3^, 10^2^, and 10^2^ copies/reaction, respectively.

### Detection of Clinical Samples Using the PCR-Tm Analysis System

**Figure 6:**
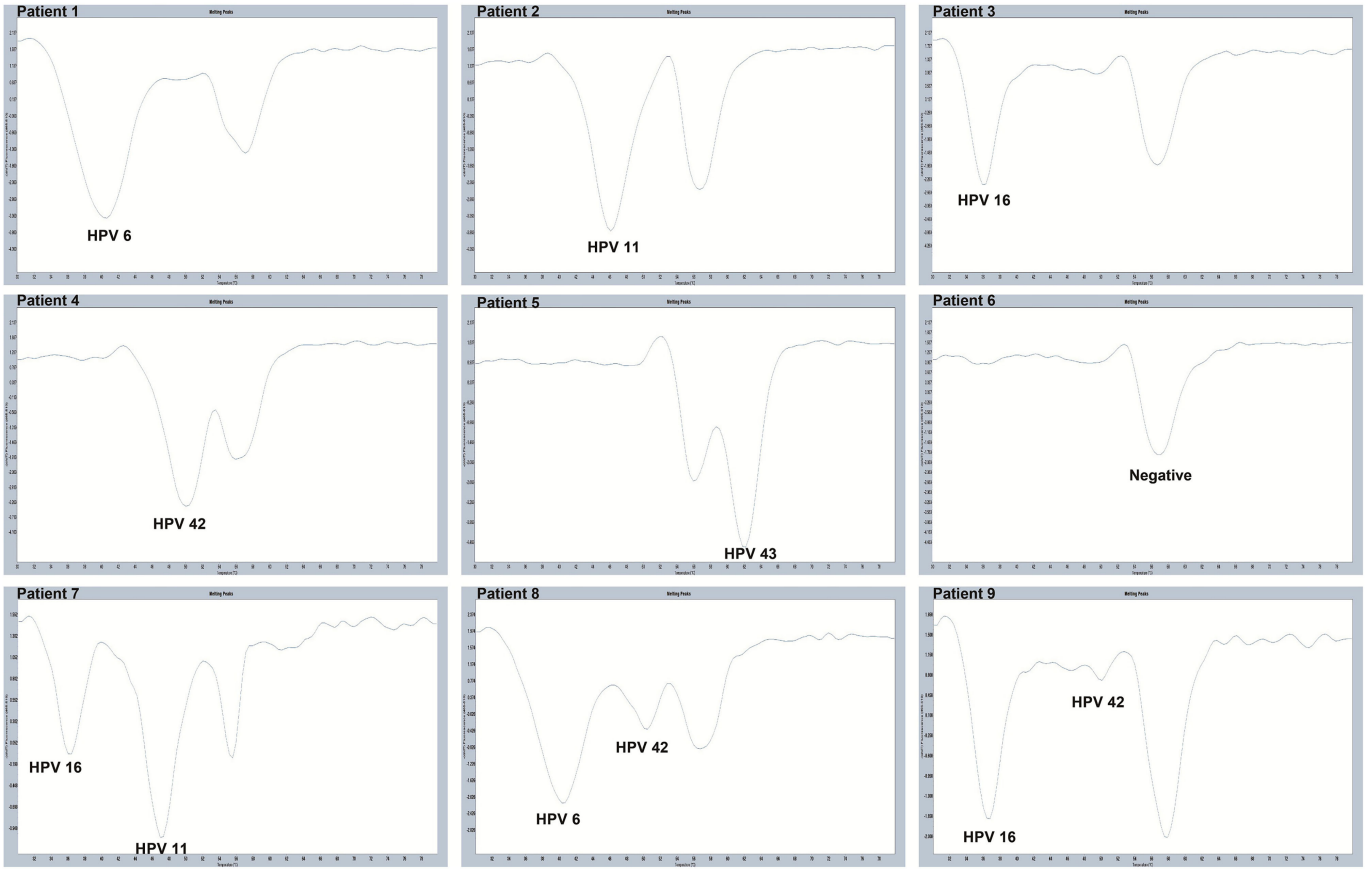
Melting curves of some clinical samples by PCR-Tm analysis. Single positive sample of HPV-6 was detected in Patient 1; single positive sample of HPV-11 was detected in Patient 2; single positive sample of HPV-16 was detected in Patient 3; single positive sample of HPV-42 was detected in Patient 4; single positive sample of HPV-43 was detected in Patient 5; negative sample was detected in Patient 6; multiple positive samples of HPV-16 and HPV-11 were detected in Patient 7; multiple positive samples of HPV-6 and HPV-42 were detected in Patient 8; and multiple positive samples of HPV-16 and HPV-42 were detected in Patient 9.

All 280 samples were tested using the PCR-Tm analysis technique and a flow fluorescence hybridization assay. All the positive samples by PCR-Tm analysis were validated using Sanger sequencing. Most of the 160 samples, including 60 HPV-other type positive and 100 HPV-negative samples tested by flow fluorescence hybridization assay, were negative by PCR-Tm analysis, except for the two HPV-other type positive samples by flow fluorescence hybridization assay; one was HPV-6 positive and one was HPV-16 positive, both by PCR-Tm analysis (Table 3). For the 120 remaining samples, we detected 25 HPV-6, 12 HPV-11, 21 HPV-16, 18 HPV-42, 25 HPV-43, and 19 multiple HPV infections by PCR-Tm analysis (Figure 6). We did Sanger sequencing for all the same samples as we analyzed with 2D-PCR. Our evaluation showed 92.6% sensitivity and 94.6% specificity for the 2D-PCR method compared to the Sanger Sequencing among the whole 280-sample dataset. However, for the flow fluorescence hybridization assay, there were only 10 multiple HPV infections detected, and the other nine multiple HPV infections positive by PCR-Tm analysis were only detected as single HPV infections (Table 3). In the agreement measurements, the kappa coefficient for analysis of PCR-Tm analysis and flow fluorescence hybridization assay was 0.940 (*P* < 0.0001), and the 95% confidence interval of the kappa coefficient was 90.3–97.7% (Table 4). The observed agreement is higher than the expected agreement, which indicates the good agreement between PCR-Tm analysis and the flow fluorescence hybridization assay.

**Table 3:** Analysis of all clinical samples by PCR-Tm and flow fluorescence hybridization assay.

**HPV types**	**samples**	**PCR-Tm analysis**	**flow fluorescence hybridization assay**	**Total samples tested**
		**Positive samples**	**Positive samples**	
HPV 6	26	26	25	26
HPV 11	12	12	12	12
HPV 42	18	18	18	18
HPV 43	25	25	25	25
HPV 16	22	22	21	22
Multiple infection	19	19	10	19
HPV-other-positive	58	0	0	58
HPV-negative	100	0	0	100

**Table 4:** Agreement analysis for all samples.

**Methods**	**Agreement**	**Expected agreement**	**Kappa**	**Std. Err**.	**Z**	**Prob>Z**	**95% CI**
PCR-Tm analysis and flow fluorescence hybridization assay	96.8%	46.7%	0.940	0.019	18.064	<0.001	(90.3%,97.7%)

## Discussion

A previous study by Zhong et al. reported the rapid detection and subtyping of HPVs (HPV-6, HPV-11, HPV-16, HPV-42, HPV-43, and HPV-44) in CA using a loop-mediated isothermal amplification assay (Zhong et al., 2018). Another study by Noriyuki et al. determined the prevalence of HPV genotypes in genital warts among women in Harare, Zimbabwe (Manyere et al., 2020). Low-risk genotypes predominated at 86% prevalence, and the most prevalent genotypes were HPV-11 (47%), HPV-6 (42%), and HPV-16 (14%). We chose to detect HPV-6, HPV-11, HPV-16, HPV-42, and HPV-43 through PCR-Tm analysis.

We established a PCR-Tm analysis method to detect five HPV genotypes (HPV-6, HPV-11, HPV-16, HPV-42, and HPV-43) and HBB/D in a single closed-tube test. We designed a FAM-labeled base-quenched probe and 15 tag complement sequences, and to confirm the Tm values of each tag complement sequence, a melting curve analysis was performed. Considering the difficulty for non-professionals to judge whether there is dual infection when the Tm values between two adjacent tag complement sequences are only slightly different, we chose six tag complements, including F1 (42°C), F2 (47°C), F15 (37°C), F3 (51°C), F10 (62°C), and F7 (55°C), and then added these tags to the upstream primers of HPV-6, HPV-11, HPV-16, HPV-42, HPV-43, and HBB/D, respectively.

We optimized the PCR-Tm analysis system, including four parts, namely, the concentration of untagged primer, tagged primer, FAM probe, and Mg^2+^. To reduce the interference of competition, untagged primers must have a higher concentration than the tagged primers in the reaction. This is because all the homologous tags and the matching FAM probe will compete for the reverse complementary sequences of the tags and, thus, may interfere with the melting curve analysis and can be more prone to false positive (Zhan et al., 2020). Furthermore, since a suitable amount of probe is important for the melting curve analysis and the detection of low-concentration samples, we increased the amounts of probe to reduce competitions caused by the reverse complementary sequences of the different tags and the distraction of a highly fluorescent background. Optimization of the Mg^2+^ concentration increased the specificity of the reaction and enzyme activity, which may have increased the PCR amplification efficiency.

We validated the PCR-Tm analysis detection by assessing its sensitivity and specificity with the plasmids as templates. This method has high specificity and no cross-reactivity across the five HPV genotypes and HBB/D. The PCR-Tm analysis results detected a 6-fold infection when a mixture of five HPV plasmids and HBB/D were used as templates. The results indicated that one single closed-tube test was able to detect multiple HPV infections. Simultaneously, we observed that the depth of the melting curves differed from that of single infections, possibly due to the competition between the reverse complementary sequences of the different tags and probes. We found that the cutoff sensitivities of HPV-6, HPV-11, HPV-16, HPV-42, HPV-43, and HBB/D were 10^3^, 10^3^, 10^4^, 10^3^, 10^2^, and 10^2^ copies/reaction, respectively.

All 280 clinical samples were tested using PCR-Tm analysis and a flow fluorescence hybridization assay, and all samples testing positive by PCR-Tm analysis and/or flow fluorescence hybridization assay were validated by Sanger sequencing. We assessed the interrater agreement between the PCR-Tm analysis assay and the flow fluorescence hybridization assay, with good agreement between PCR-Tm analysis and the flow fluorescence hybridization assay. Out of a total of 280 clinical samples, 122 were positive by PCR-Tm analysis, while 120 were positive by flow fluorescence hybridization assay. Two samples tested positive for HPV-other type positive by flow fluorescence hybridization assay, but one was HPV-6 positive and the other was HPV-16 positive by PCR-Tm analysis. For the 120 samples that yielded positive results by both methods, some differences were observed among the detection results of multiple HPV infections. A total of 19 multiple HPV infection samples were positive by PCR-Tm analysis, but only 10 multiple HPV infection samples were positive by flow fluorescence hybridization assay, and the other nine samples were only detected as single HPV infections. For example, Patient 21 was positive for HPV-43 and HPV-16 by PCR-Tm analysis, while that patient was only positive for HPV-43 by flow fluorescence hybridization assay. These results indicate that compared to the flow fluorescence hybridization assay, PCR-Tm analysis is more accurate in detecting single HPV infections in clinical samples.

The PCR-Tm analysis is a single closed-tube multiplex PCR assay that combines PCR and melting curve analysis. We only reported a PCR-Tm analysis method to detect six target genes by the FAM fluorescence channel, but the detection flux can be further increased by adding more tags and using more fluorescent channels. It is expected to achieve the goal of detecting 30 target genes in a single closed-tube test using the PCR-Tm analysis assay (Zhan et al., 2020). The study by Luo et al. reported that the total approximate cost for one reaction of quantitative real-time PCR (Q-PCR) is US$0.055, including costs of primers, probe, buffer, dNTPs, and Taq DNA polymerase (Luo et al., 2009). Compared with Q-PCR, the cost of PCR-Tm analysis in forward and reverse primers is significantly higher. It was found that 5 OD of primer can detect approximately 2,000 DNA samples. If calculated according to 30 pairs of HPV primers, it costs approximately US$1.42 per sample. However, the cost of the flow fluorescence hybridization assay (Shanghai Topview Life Technology Co., Ltd.) is approximately US$21.52 per sample. Therefore, PCR-Tm analysis is more economical than the flow fluorescence hybridization assay.

Currently, a large number of tools are available for genotyping a greater diversity of HPV types and their variants. Among them, the second-generation hybrid capture method (HC2) is easy to operate, suitable for large-scale population screening, and the results are objective and stable, but it cannot perform HPV individual genotyping (Munoz et al., 2004). Gene chip technology detects quickly and can perform genotyping, but it also has the disadvantages of high testing costs and high requirements for testing equipment and personnel (Clifford et al., 2003). The SPF10-INNO-LiPA assay can amplify up to 43 different genotypes and provide type-specific genotype information for 25 different HPV genotypes simultaneously by PCR amplification of a 65 bp region of the conserved *L1* gene and reverse line blot hybridization to identify specific HPV types (van Hamont et al., 2006). However, this assay is an open-tube PCR method that has contamination risks, which could lead to false results, and require additional product identification equipment and complex processing procedures. Luminex suspension array technology, which combines PCR with hybridization to fluorescence-labeled polystyrene bead microarrays, has also been used for the clinical detection of HPV genotypes (Syrjanen et al., 2012). The flow fluorescence hybridization assay is based on the Luminex100 technology platform and the Luminex100 multifunctional flow analyzer. Both methods, PCR-Tm analysis and the flow fluorescence hybridization assay, have no need for significant technical skills and could detect and identify a variety of target genes with high throughput (Chen et al., 2009; Zhan et al., 2020). However, some disadvantages of the flow fluorescence hybridization assay are that it requires additional equipment, the steps are cumbersome, the tube needs to be open during the process, the detection time is long, and it is easy to incur laboratory contamination. This PCR-Tm analysis assay is simple in operation because it is just an assay combining PCR and melting curve analysis. Moreover, since PCR-Tm analysis can detect and identify a variety of target genes with high throughput in a single tube and under closed-tube conditions, it can avoid laboratory contamination and does not require any additional instrumentation to identify PCR products. It is suitable for large-scale CA screening and diagnosis, and it is also very suitable for various grassroot hospitals to carry out. It has strong clinical application value and potential for diagnostic transformation and reduces the time and stress burden on patients. PCR-Tm analysis has the primary advantages of high-throughput, low running cost, simple equipment and operation, and allows early screening and prevention of CA. For clinical laboratories, this technique could be valuable.

There were some limitations to our study. First, the number of clinical samples was small, and it is necessary to expand the sample size for further research. Second, we only detected five HPV genotypes, which do not have much value for clinical application. However, this method can detect more genotypes by increasing the number of fluorescent channels.

## Conclusion

The PCR-Tm analysis assay enabled the detection of five HPV genotypes (HPV-6, HPV-11, HPV-16, HPV-42, and HPV-43), including single and multiple infections, with a single closed-tube test combining PCR and melting curve, thus allowing for early screening of CA.

## Data Availability Statement

The original contributions presented in the study are included in the article/Supplementary Material, further inquiries can be directed to the corresponding author/s.

## Ethics Statement

The studies involving human participants were reviewed and approved by the Ethics Committee of Shanghai Tenth People's Hospital (No. 20KT141). Written informed consent for participation was not required for this study in accordance with the national legislation and the institutional requirements.

## Author Contributions

WL, HS, and HL conceived and designed the experiments. LW, XW, and WW performed experiments. JZ, YC, and XG collected clinical samples. WL and LW wrote the manuscript. All authors read and approved the final manuscript.

## Funding

This study was supported by grants from the Outstanding Academic Leaders Plan of Shanghai (Grant No. 2018BR07) and the Shanghai Health and Family Planning Commission Clinical Research Project for Health Industry (Grant No. 202140147).

## Conflict of Interest

The authors declare that the research was conducted in the absence of any commercial or financial relationships that could be construed as a potential conflict of interest.

## Publisher's Note

All claims expressed in this article are solely those of the authors and do not necessarily represent those of their affiliated organizations, or those of the publisher, the editors and the reviewers. Any product that may be evaluated in this article, or claim that may be made by its manufacturer, is not guaranteed or endorsed by the publisher.
