# Supplementary material for: Detection of Five Types of HPV Genotypes Causing Anogenital Warts (Condyloma Acuminatum) Using PCR-Tm Analysis Technology
Source: Front Microbiol. 2022 May 17;13:857410. doi: 10.3389/fmicb.2022.857410 (PMC9152731; doi:10.3389/fmicb.2022.857410)
Supplement: Supplementary file 1 [file Table_1.DOC]

Supplementary Table 1: Primers information

| Genotype | Primer sequence (5′–3′) | Genome position | Length of the amplimer |
| --- | --- | --- | --- |
| HPV-6 (GI:60955) | F:AGACGTGCTAATTCGGTGCT | E6:394-414 | 134 bp |
|  | R:TTGTCCAGCAGTGTAGGCAG | E6:528-509 |  |
| HPV-11 (GI:333026) | F:TTGCAGGAATGCACTGACCA | E6:200-219 | 233 bp |
|  | R:ACGGCTTGTGACACAGGTAA | E6:432-413 |  |
| HPV-16 (GI:333031) | F:TAATTCACAGGCAAAAATTGTAAAGG | E1:2049-2074 | 98 bp |
|  | R:ATTTTATCCATTGACTCATACTCATTTGT | E1:2146-2118 |  |
| HPV-42 (GI:333211) | F:GATGTAGGGTTTGGGGCACT | L1:6431-6450 | 194 bp |
|  | R:GCGCCAGCCCTATTAAACAA | L1:6624-6605 |  |
| HPV-43 (GI:40804474) | F:AGCGTTTAGTCTGGGGATGC | L1:6037-6056 | 141 bp |
|  | R:TATCTTGTCCCGGCGATGTG | L1:6177-6158 |  |
